# Mitral Stenosis in the Multimodality Imaging Era: Pitfalls, Stress Echocardiography, and Integrated Therapeutic Assessment

**DOI:** 10.3390/diagnostics16142285

**Published:** 2026-07-22

**Authors:** Barbara Pala, Mariagrazia Piscione, Dario Gaudio, Paola Gualtieri, Mario Laudazi, Simone Steffani, Francesco Giuseppe Garaci, Marco Alfonso Perrone, Laura Di Renzo

**Affiliations:** 1 PhD School of Applied Medical-Surgical Sciences, Tor Vergata University of Rome, Via Montpellier 1, 00133 Rome, Italy; barbara.pala93@gmail.com; 2 UOC Cardiologia Ospedale, IDI-IRCCS, Via Monti di Creta 104, 00167 Rome, Italy; 3 Santissima Annunziata Hospital, Via dei Vestini, 66100 Chieti, Italy; mariagraziapiscione@gmail.com; 4 Fondazione Policlinico Campus Biomedico, Via Alvaro del Portillo 200, 00128 Rome, Italy; dario.gaudio@unicampus.it; 5 Section of Food Science, Clinical Nutrition and Pharmaceutical Sciences, Department of Biomedicine and Prevention, Tor Vergata University of Rome, Via Montpellier 1, 00133 Rome, Italylaura.di.renzo@uniroma2.it (L.D.R.); 6 Diagnostic Imaging Division, Department of Biomedicine and Prevention, Tor Vergata University of Rome, Via Cracovia 50, 00133 Rome, Italy; mariolauda@gmail.com (M.L.); simone.soldato94@gmail.com (S.S.); francesco.garaci@uniroma2.it (F.G.G.); 7 Division of Cardiology and CardioLab, Department of Clinical Sciences and Translational Medicine, Tor Vergata University of Rome, Via Montpellier 1, 00133 Rome, Italy

**Keywords:** mitral stenosis, rheumatic disease, degenerative mitral stenosis, mitral annular calcification, multiparametric assessment

## Abstract

Mitral stenosis (MS) remains a clinically relevant condition worldwide, with rheumatic and degenerative aetiologies contributing to a broad spectrum of disease. Accurate assessment of MS severity is essential for clinical decision-making but is often challenged by technical limitations, complex hemodynamic interactions, and the heterogeneous anatomical characteristics of different MS aetiologies. This review aims to provide a comprehensive overview of the contemporary multimodality imaging assessment of MS, with particular emphasis on the pitfalls of conventional transthoracic echocardiography (TTE), the incremental value of advanced imaging modalities, and their integration into diagnostic and therapeutic decision-making. A targeted narrative review of the literature was conducted focusing on multimodality imaging approaches and their integration into clinical practice. Particular emphasis was placed on TTE parameters, three-dimensional (3D) TTE, stress echocardiography, transoesophageal echocardiography (TOE), cardiac computed tomography (cCT), and cardiac magnetic resonance (CMR), highlighting their complementary roles in anatomical characterization, hemodynamic assessment, procedural planning, and clinical decision-making. Conventional 2D TTE remains the cornerstone for MS evaluation; however, widely used parameters such as mean transmitral gradient (TMG) and pressure half-time (PHT) are highly load-dependent and may lead to misclassification of disease severity in the presence of altered hemodynamic conditions (e.g., tachycardia, atrial fibrillation (AF), or reduced cardiac output). Although direct planimetry remains the anatomical reference standard, its accuracy may be limited by operator dependency, calcification, and complex valve geometry. In this context, three-dimensional TTE (3D TTE) provides incremental value by enabling more accurate visualization of the mitral valve (MV) orifice and improving measurement reproducibility. Stress TTE plays a key role in patients with discordant symptoms or borderline resting findings by unmasking clinically significant disease during exercise. In selected patients, TOE, cCT, and CMR provide complementary information for the evaluation of complex valve morphology, mitral annular calcification (MAC), ventricular remodelling, and procedural planning, particularly in degenerative mitral stenosis (DMS) and candidates for transcatheter interventions. The evaluation of MS requires an integrated, multiparametric, multimodality imaging approach. Combining conventional TTE with stress imaging and complementary advanced modalities improves diagnostic accuracy, facilitates patient selection for intervention, and supports individualized clinical decision-making, particularly in patients with complex anatomy or discordant imaging findings.

## 1. Introduction

Mitral stenosis (MS) remains a clinically relevant valvular heart disease despite its changing epidemiology over recent decades [1]. While rheumatic disease continues to be the leading cause worldwide, degenerative and calcific forms are increasingly encountered in ageing populations, often in association with multiple comorbidities [1]. Regardless of aetiology, MS is characterized by an obstruction to left ventricular (LV) inflow, leading to elevated left atrial (LA) pressure, pulmonary hypertension (PH), and progressive LV function limitation [1].

The non-invasive assessment of MS has progressively evolved over time, largely replacing invasive hemodynamic evaluation. Early insights into mitral valve (MV) dynamics were provided by Inge Edler and Carl Hellmuth Hertzusing M-mode echocardiography (TTE) [2]. The introduction of two-dimensional imaging by William L. Henry and colleagues in the mid-1970s enabled direct visualization of the mitral orifice and planimetric measurement of valve area, reducing the reliance on cardiac catheterization [3]. This shift was further supported by the demonstration from Håkon Holen of a strong correlation between Doppler-derived and invasive transmitral gradients (TMGs) [4]. Finally, the work of Liv Hatle in 1979 introduced pressure half-time (PHT) as a simple and widely adopted method for estimating mitral valve area (MVA) [5]. Despite these major advances, the TTE assessment of MS remains subject to several limitations.

Traditional parameters, including MVA, TMGs, and PHT, are widely used in clinical practice but are influenced by several hemodynamic and technical factors [6]. Heart rate, loading conditions, LA and LV compliance, and concomitant valvular lesions may all significantly affect these measurements, potentially leading to discordant or misleading results. Therefore, the evaluation of MS severity remains particularly challenging in borderline cases or in the presence of incongruent TTE findings and clinical presentation [7].

In this context, reliance on a single parameter may be inadequate, and a comprehensive, multiparametric approach is essential. Advances in imaging, including three-dimensional TTE (3D TTE) and stress testing, have provided additional tools to better characterize valve anatomy and assess the functional impact of the stenosis under dynamic conditions [8]. 3D imaging allows a more accurate planimetric assessment of the MV orifice, overcoming some of the geometric limitations of two-dimensional techniques, while exercise or pharmacological stress testing can unmask clinically relevant hemodynamic changes not evident at rest [8].

Despite these advances, important pitfalls in the assessment of MS persist, and a standardized, integrated diagnostic approach is still lacking in many clinical settings. The aim of this review is to critically appraise the limitations of conventional TTE parameters, to highlight the role of advanced imaging techniques, and to propose a practical framework for the comprehensive evaluation of MS severity.

Unlike previous reviews focusing on individual imaging modalities or specific etiologies of MS, the primary aim of this review is to provide a practical multimodality imaging framework for the diagnostic assessment of this valve pathology. Particular emphasis is placed on recognizing the limitations and pitfalls of conventional TTE parameters, understanding the complementary role of stress TTE, cardiac computed tomography (cCT), and cardiac magnetic resonance (CMR), and integrating these imaging modalities into a practical diagnostic workflow that supports clinical decision-making in both rheumatic and degenerative mitral stenosis (DMS). Therapeutic considerations are discussed only insofar as they influence imaging selection and interpretation.

## 2. Literature Search Strategy

This narrative review provides a practical overview of contemporary multimodality imaging assessment of MS, including rheumatic, degenerative, and congenital aetiologies.

A targeted search of PubMed/MEDLINE was performed on 25 May 2026, for English-language articles published up to May 2026, using combinations of the terms “mitral stenosis,” “rheumatic mitral stenosis,” “degenerative mitral stenosis,” “mitral annular calcification,” “echocardiography,” “stress echocardiography,” “mitral valve area,” “Wilkins score,” “percutaneous mitral commissurotomy,” “cardiac computed tomography,” “cardiac magnetic resonance,” and “multimodality imaging.” Reference lists of relevant articles and current international guidelines were also screened to identify additional publications.

Priority was given to original clinical studies, landmark publications, and contemporary guideline recommendations addressing the diagnosis, hemodynamic assessment, procedural planning, and management of MS; classical studies were included when essential to understanding the development of key imaging techniques. Case reports, conference abstracts, editorials, and non-English publications were excluded. As this is a narrative rather than systematic review, no formal PRISMA methodology or quantitative synthesis was applied.

## 3. Aetio-Pathophysiology of Mitral Valve Stenosis

### 3.1. Aetiology of Rheumatic Mitral Stenosis

Rheumatic mitral stenosis (RMS) represents by far the most common form of MV obstruction worldwide [9,10]. It originates from acute rheumatic fever, a delayed autoimmune sequela of untreated or inadequately treated pharyngitis caused by group A β-hemolytic *Streptococci* in genetically predisposed individuals [11]. This systemic inflammatory disease may present with extracardiac manifestations such as migratory polyarthritis, Sydenham chorea, erythema marginatum, and subcutaneous nodules. In the acute phase, pancarditis—particularly myocarditis—can occur and may be life-threatening, whereas chronic sequelae predominantly involve the cardiac valves, most frequently leading to MS [11]. The underlying mechanism involves molecular mimicry: antibodies and T lymphocytes directed against streptococcal antigens (notably the M protein) cross-react with myocardial and valvular extracellular matrix proteins, triggering an inflammatory response characterized by the formation of Aschoff bodies within the myocardium and along the valvular endocardium [11]. During the healing phase, this inflammatory process evolves into fibrotic remodeling, resulting in leaflet thickening, commissural fusion, chordal shortening and fusion, and progressive calcific deposition, ultimately leading to the characteristic anatomical and functional features of RMS [11].

### 3.2. Pathophysiology of Rheumatic Mitral Stenosis

RMS is characterized by a progressive reduction in the MVA [12]. More in detail, the obstruction to LV filling may occur at three different levels: at a pre-orificial level, where the effective MVA may vary independently of the anatomical stenotic orifice; at the valvular level itself, due to leaflet rigidity and/or commissural fusion; and at the subvalvular level, as a consequence of chordal thickening, shortening, and retraction [12]. This multilevel obstruction generates a persistent TMG throughout diastole, leading to a chronic elevation in LA pressure, which is transmitted retrogradely to the pulmonary veins and capillaries, promoting fluid transudation into the interstitium and alveoli [12]. The progressive increase in pulmonary venous pressure results in passive PH, initially affecting veins, then capillaries, and subsequently arterioles [12]. The extent of pulmonary involvement may be significantly modulated by underlying pulmonary diseases, such as chronic obstructive pulmonary disease or pulmonary fibrosis [13].

Furthermore, LA compliance plays a crucial role in lessening pressure impact: the LA pressure waveform is characterized by an a wave, reflecting atrial contraction, and a v wave, reflecting atrial filling during ventricular systole [13]. In a highly compliant LA, a given increase in volume produces only a modest rise in pressure and a relatively small v wave, whereas in a stiff LA even minimal volume changes result in a marked pressure increase and a prominent v wave. Recent evidence suggests that reduced LA compliance is associated with a higher likelihood of developing PH [13]. Over time, sustained pulmonary venous hypertension induces structural remodeling of the pulmonary vasculature, initially characterized by wall thickening, followed by smooth muscle cell hyperplasia, and, in more advanced stages, by marked intimal proliferation and concentric medial fibrosis [14]. The consequent increase in right ventricular (RV) afterload leads to RV dilation and dysfunction, often accompanied by tricuspid annular enlargement, ultimately contributing to the development of right-sided heart failure [14].

### 3.3. Aetiology of Degenerative Mitral Stenosis

DMS is an increasingly recognized cause of MS, particularly among elderly women, and is most related to extensive mitral annular calcification (MAC) [15]. MAC is a complex, active, and multifactorial process rather than a passive consequence of aging. Histopathological studies have demonstrated that MAC is characterized by lipid deposition, chronic inflammation, fibrosis, neoangiogenesis, and progressive calcium accumulation within the mitral annulus and adjacent leaflet tissue [15]. As the disease progresses, calcified and necrotic tissue becomes embedded within a collagen-rich matrix infiltrated by inflammatory cells, with evidence of osteogenic differentiation, bone morphogenetic protein activation, and even lamellar bone formation in advanced stages [16]. Similar to aortic valve calcification and atherosclerosis, MAC appears to develop through an initial phase of endothelial injury and inflammation, followed by a propagation phase driven by osteogenic signalling and dysregulation of calcium–phosphate metabolism [17].

MAC shares several cardiovascular risk factors with systemic atherosclerosis, including hypertension, diabetes, obesity, dyslipidaemia, and smoking, and frequently coexists with coronary and vascular calcification [18]. However, important differences exist, particularly the higher prevalence in women and the stronger association with triglyceride-related metabolic pathways rather than LDL-related mechanisms [19]. Chronic kidney disease and mineral bone disorders also play a major role in MAC development, as abnormalities in phosphate handling, fibroblast growth factor-23, fetuin-A, and calcium–phosphate homeostasis promote ectopic calcification [18]. Inflammation further contributes to disease progression, with inflammatory biomarkers such as C reactive protein and interleukin-6 being associated with incident MAC and its progression [20]. More recently, positron emission tomography studies using ^18^F-FDG and ^18^F-sodium fluoride have confirmed the presence of active inflammation and ongoing calcification activity within the mitral annulus, suggesting the existence of a self-perpetuating cycle of inflammation and progressive calcification that may drive disease progression over time (Table 1) [20].

These pathophysiological differences translate into distinct clinical trajectories and management pathways. As a matter of fact, patients with DMS are typically older and carry a substantially higher burden of comorbidities than those with RMS, with a natural history shaped more by systemic frailty and competing morbidities than by the valve lesion alone [19]. As a result, non-cardiac mortality frequently competes with valve-related mortality in this population, complicating risk stratification and the interpretation of prognostic parameters such as the TMG [6,7]. Procedural options also diverge markedly: percutaneous mitral commissurotomy (PMC), effective in RMS due to preserved commissural anatomy, is generally unsuitable in DMS because of annular calcification and the absence of fusible commissures [11,17]. Conventional surgical replacement in DMS carries increased technical complexity and risk related to annular involvement and patient comorbidity burden—a pattern also observed when MAC coexists with other valve interventions such as transcatheter aortic valve replacement [18]—prompting growing interest in dedicated transcatheter approaches for calcific mitral disease as alternatives in patients at high surgical risk [11].

### 3.4. Aetiology and Pathophysiology of Congenital Mitral Valve Diseases

Congenital MV diseases include a heterogeneous spectrum of developmental abnormalities affecting the mitral leaflets, chordae tendineae, PMs, or mitral annulus [1]. These defects arise from anomalies in the complex embryological processes governing atrioventricular valve formation, including endocardial cushion development, epithelial–mesenchymal transition, extracellular matrix remodelling, and PM differentiation [1]. Even if they may occur as isolated defects, they are frequently associated with other congenital heart diseases, such as atrioventricular septal defects, LVOT obstruction, coarctation of the aorta, and complex left-sided obstructive lesions [1].

The anatomical abnormalities may result in MS, MR, or a combination of both [1]. Congenital mitral stenosis is most commonly caused by leaflet thickening, commissural fusion, supramitral ring, parachute MV, hammock MV, or abnormal chordal insertion, all of which restrict leaflet opening and impair diastolic filling of the LV [1]. The resulting increase in LA pressure leads to progressive LA enlargement, pulmonary venous hypertension, and, in severe cases, PH and RV dysfunction [1].

## 4. Echocardiographic Assessment of Mitral Stenosis

### 4.1. Morphological Assessment in Mitral Stenosis

#### 4.1.1. Morphology in Rheumatic Mitral Stenosis

Morphological assessment represents a cornerstone in the evaluation of MS, as it provides essential information for diagnosis, severity grading, therapeutic planning, and procedural feasibility [21].

**Figure 1:**
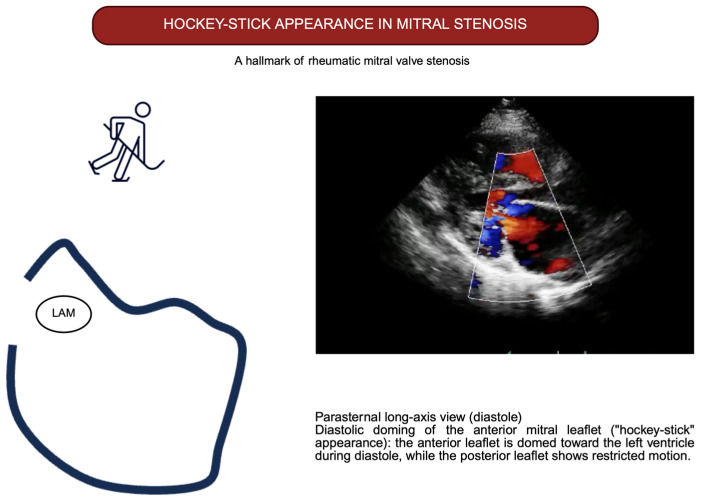
Hockey-stick appearance in mitral stenosis.

**Figure 2:**
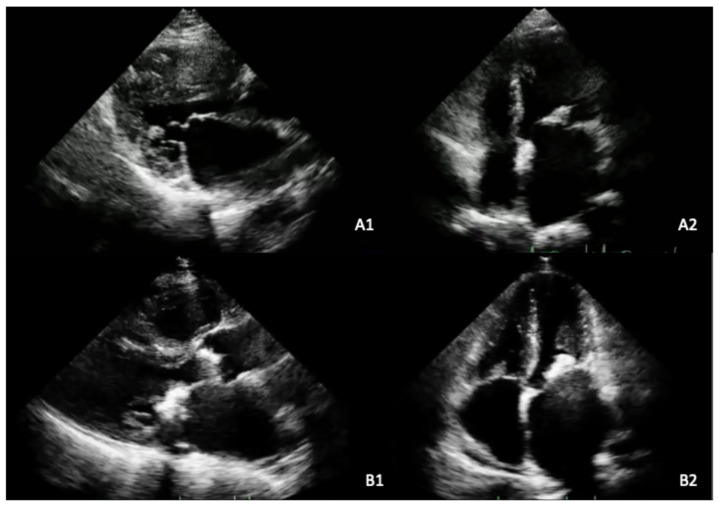
Representative TTE images of RMS obtained from parasternal long-axis (**A1**,**B1**) and apical four-chamber (**A2**,**B2**) views. The images illustrate typical rheumatic morphological features, including leaflet thickening, restricted leaflet mobility, commissural fusion, and varying degrees of left atrial enlargement. Panels (**A1**,**A2**) demonstrate a relatively more favorable valvular morphology with partially preserved leaflet opening despite the presence of chordal calcification, whereas panels (**B1**,**B2**) show more advanced structural remodeling with marked leaflet thickening, severe subvalvular involvement, reduced mitral valve opening area, and extensive calcification of the anterior mitral leaflet, consistent with severe rheumatic mitral stenosis.

In RMS, commissural fusion represents the hallmark lesion and should be carefully assessed during TTE examination [22]. From a pathophysiological perspective, commissural fusion likely develops preferentially in leaflet segments with reduced diastolic excursion during the chronic healing phase of rheumatic inflammation [23]. Fusion may symmetrically involve both the anterolateral and posteromedial commissures, although asymmetric fusion can also occur and has major implications for PMC [23]. Indeed, balloon dilation exerts its effect mainly on the fibrotic commissural scar, which represents the site of least mechanical resistance; therefore, commissural morphology strongly influences the likelihood of successful commissural splitting and post-procedural valve area gain [24] (Figure 1 and Figure 2).

Leaflet morphology also plays a crucial role. The stenotic rheumatic valve progressively acquires a funnel-shaped configuration extending from the annulus toward the leaflet tips. The anterior mitral leaflet (AML), being longer and more mobile, is often initially affected at its free margin, while the basal pars liscia may remain relatively preserved in earlier stages. In contrast, the posterior mitral leaflet (PML), which is shorter and less mobile, frequently becomes retracted, rigid, and hypomobile, contributing to the typical diastolic doming appearance of the AML [23]. Regardless of the treatment strategy employed, the main mechanism underlying MVA enlargement remains separation of the fused leaflet margins [23]. Consequently, preservation of AML flexibility is associated with greater post-interventional MVA increase, whereas extensive fibrosis and calcification predict less favourable hemodynamic results [24].

Assessment of the subvalvular apparatus is equally important, since chordal thickening, fusion, and shortening generally parallel leaflet involvement. It is uncommon to observe thin, pliable leaflets coexisting with severe subvalvular disease [25]. However, evaluation of the subvalvular apparatus remains challenging with 2D-TTE, and no true gold standard currently exists. In clinical practice, preserved subvalvular anatomy is suggested when papillary muscles remain distinguishable from the chordae tendineae and the chordae maintain adequate length, generally greater than 10 mm [25].

Calcification should also be systematically assessed because its location has important therapeutic implications. Commissural calcification may significantly reduce the efficacy and safety of PMC (this concept will be further developed and explained in the next paragraphs), whereas calcification confined to the leaflet body may sometimes be surgically debrided. From an imaging standpoint, calcifications appear as highly echogenic structures associated with acoustic shadowing, frequently obscuring deeper anatomical structures and complicating valve assessment [26].

#### 4.1.2. Morphology in Degenerative Mitral Stenosis

DMS is characterized by progressive MAC involving the annulus and basal portions of the leaflets, while commissural fusion is typically absent and leaflet tips often remain relatively mobile [27]. As calcification progresses, a rigid tunnel-like inflow tract develops, with the greatest narrowing occurring at the annular base rather than at the leaflet tips [27]. TTE grading of MAC is commonly based on the circumferential extension of calcification along the posterior mitral annulus in the parasternal short-axis (PSAX) view and may be classified as mild when involving less than one-third of the annular circumference, moderate when extending up to two-thirds, and severe when involving more than two-thirds [27] (Figure 2). In addition to this conventional TTE grading system, a more comprehensive classification has recently been proposed by the Heart Valve Collaboratory in a position statement published in JACC Cardiovascular Imaging [28]. The document emphasized that previous studies used highly heterogeneous definitions of MAC severity based on either TTE or cCT. To overcome the limited comparability between studies, the proposed classification integrates multimodality imaging, including TTE and TOE together with cCT, with particular emphasis on a dedicated cCT-based MAC score [28]. It will be further discussed and analyzed.

Unlike RMS, the residual orifice in DMS often assumes a crescent-like configuration due to preservation of commissural opening [29]. Standard TTE grading systems may underestimate disease complexity because they frequently fail to account for extra-annular extension of calcification, which may substantially contribute to valve dysfunction and procedural complexity. In this context, three-dimensional transoesophageal echocardiography (3D TOE) is often superior to conventional TTE imaging for defining calcific distribution and direct planimetry of the residual orifice. Morphological assessment in DMS should additionally evaluate associated mitral regurgitation (MR) related to leaflet restriction or localized flail, as well as potential complications such as thrombus formation or caseous MAC, the latter representing liquefactive necrosis within the calcified annulus that may mimic cardiac masses or abscesses on imaging [27].

### 4.2. Mean Transmitral Gradient

The mean TMG represents one of the most widely used Doppler-derived parameters for the hemodynamic assessment of MS. It is obtained using continuous-wave Doppler across the MV and reflects the average pressure difference between the LA and LV during diastole [30,31].

However, interpretation of the TMG requires careful integration with anatomical and clinical findings, as TMG are highly flow-dependent and may not directly reflect the true anatomical severity of stenosis [23]. According to the simplified Bernoulli equation, transmitral velocity and gradient increase exponentially with transvalvular flow [23]. Consequently, several physiological and pathological conditions may substantially influence TMG independently of MVA. Tachycardia shortens diastolic filling time and increases transmitral flow velocity, thereby leading to higher gradients for a given stenotic orifice. Similarly, conditions associated with increased cardiac output, such as anaemia, fever, pregnancy, hyperthyroidism, or exercise, may significantly elevate the TMG despite only moderate anatomical MS [23]. Conversely, low-flow states may result in deceptively low gradients even in severe MS [23].

Cardiac rhythm also plays a major role in gradient interpretation. In atrial fibrillation (AF), beat-to-beat variability in diastolic filling results in marked fluctuations in TMGs, requiring averaging over multiple cardiac cycles for accurate assessment [23]. Furthermore, concomitant MR may increase transmitral flow and artificially elevate the TMG independently of MS severity [23].

Moreover, the relationship between TMG and MS severity differs substantially between RMS and DMS. In RMS, there is a more direct relationship between MVA and TMG [23]. In contrast, DMS is characterized by a rigid tunnel-like inflow tract involving the mitral annulus and leaflet bases, producing less abrupt flow acceleration and often lower gradients for a comparable anatomical MVA [27]. Elderly patients with DMS frequently exhibit reduced LV compliance and concomitant diastolic dysfunction, factors that may further modify transmitral hemodynamics and contribute to underestimation of stenosis severity [23].

For these reasons, no single TMG threshold should be interpreted in isolation. Current guidelines generally consider a mean gradient ≥5 mmHg suggestive of significant MS under resting conditions, but it should not be considered a stand-alone severity threshold [7]. In addition, increasing evidence suggests that higher resting gradients are associated with worse outcomes, particularly in DMS, where a mean TMG exceeding 8–10 mmHg has been linked to adverse prognosis [32]. Overall, the TMG should be regarded as an integrated hemodynamic marker reflecting the interaction between anatomical obstruction, transvalvular flow, and ventricular-atrial compliance rather than a pure surrogate of MVA alone.

### 4.3. Planimetry

**Figure 3:**
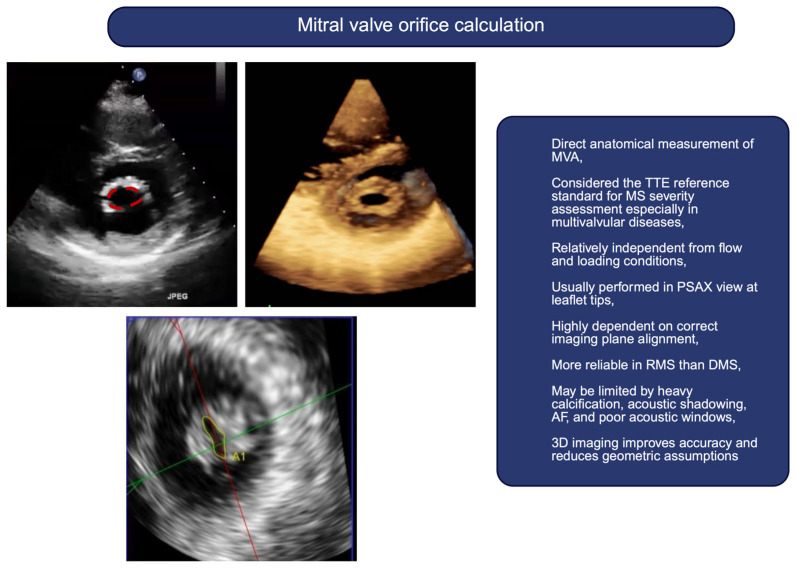
2D and 3D TTE planimetry of the MVA in PSAX view at the level of the leaflet tips. Direct tracing of the mitral orifice (red dashed contour) allows anatomical quantification of MVA and represents the TTE reference standard for MS severity assessment. Although relatively independent from flow conditions, planimetry accuracy is highly dependent on optimal imaging plane alignment and may be limited by calcification, acoustic shadowing, atrial fibrillation, and irregular valve geometry. Abbreviations: AF: atrial fibrillation, DMS: degenerative mitral stenosis, MVA: mitral valve area, PSAX: parasternal short axis view, RMS: rheumatic mitral stenosis, TTE: trans-thoracic echocardiography.

Direct planimetry of the mitral valve orifice represents the most direct TTE method for anatomical assessment of MVA, especially in multivalvular disease, and is generally considered the reference standard for the evaluation of MS severity [23]. Unlike Doppler-derived parameters, planimetry is relatively independent of transvalvular flow conditions and therefore provides a more direct estimation of the anatomical stenotic orifice [23]. Traditionally, 2D-TTE planimetry is performed in the PSAX view at the level of the leaflet tips during diastole, aiming to identify the smallest functional orifice or in the subcostal view [23] (Figure 3). When adequate acoustic windows are available, 3D TTE is generally sufficient for anatomical assessment and MVA planimetry. Conversely, 3D TOE provides higher spatial resolution and may be preferred in patients with suboptimal TTE image quality, complex valve anatomy, extensive calcification, or when detailed pre-procedural assessment is required [22,29]. In RMS, planimetry is usually feasible and reliable because the stenotic orifice is typically located at the leaflet tips [23]. However, accurate measurement requires meticulous alignment of the imaging plane perpendicular to the stenotic orifice, since oblique imaging may significantly overestimate the effective MVA [23]. Additional limitations include poor acoustic windows, heavy calcification, AF, and marked leaflet distortion [23]. Gain settings and image quality may also substantially influence contour delineation and measurement reproducibility [23].

The assessment becomes considerably more challenging in DMS. The greatest narrowing typically occurs at the annular base rather than at the leaflet tips, generating a tunnel-like and frequently crescent-shaped inflow orifice. In this setting, conventional 2D planimetry performed at the leaflet tip level may substantially overestimate the true functional MVA [27]. Furthermore, severe calcification often produces prominent acoustic shadowing and reverberation artifacts that obscure the mitral inflow tract and impair accurate delineation of the stenotic orifice. 3D TTE improves anatomical assessment of MS primarily through multiplanar reconstruction (MPR). Unlike single-plane 2D imaging, MPR allows simultaneous alignment of orthogonal cropping planes across the full 3D dataset, enabling the operator to systematically identify and trace the true smallest anatomical orifice regardless of its spatial orientation, rather than relying on a single, potentially oblique, imaging plane [27]. This capability is particularly valuable in three clinical scenarios: asymmetric rheumatic valves, where commissural fusion is often non-uniform and the stenotic orifice may not lie in a standard imaging plane; crescent-shaped calcific stenoses typical of DMS, where the orifice is irregular and eccentrically located at the annular/basal level rather than at the leaflet tips; and tunnel-like geometries, where the narrowest point may vary in position along the length of the inflow tract, making single-plane 2D measurement particularly prone to error. MPR is also central to pre-procedural planning, allowing precise characterization of orifice shape, calcium distribution, and spatial relationship with adjacent structures such as the LVOT.

It is important to distinguish the anatomical valve area, obtained by direct tracing of the orifice using planimetry or MPR, from the effective valve area, derived from Doppler-based flow calculations such as PHT and the continuity equation. The two do not always coincide: flow convergence, non-planar orifice geometry, and the presence of multiple sub-orifices—as can occur in heavily calcified or tunnel-like DMS anatomy—may cause the effective orifice, as “seen” by the flow field, to differ from the anatomical orifice identified by imaging. This distinction reinforces the rationale for a multiparametric approach rather than reliance on any single anatomical or Doppler-derived measurement.

3D-TTE and 3D-TOE play complementary rather than interchangeable roles. 3D-TTE is non-invasive, widely available, and sufficient in most patients with adequate acoustic windows, making it a reasonable first-line 3D modality; however, its spatial and temporal resolution remain lower than TOE, and image quality can be significantly degraded by poor acoustic windows, obesity, or lung interposition. 3D-TOE offers higher spatial resolution and superior near-field visualization of the MV apparatus and is generally preferred in patients with suboptimal TTE windows, complex or asymmetric valve anatomy, extensive calcification, or when detailed pre-procedural planning is required (e.g., before PMC or transcatheter intervention) [22,29]. Its limitations include semi-invasiveness, the need for sedation or general anaesthesia, and continued vulnerability to acoustic shadowing from dense annular or leaflet calcification, which may still obscure orifice delineation even with MPR. Consequently, in heavily calcified DMS, 3D TTE should be interpreted alongside cCT, which provides superior spatial resolution for calcium quantification and is less affected by acoustic artifact [27] (Table 2).

### 4.4. Pressure Half Time

PHT is one of the most widely used Doppler-derived methods for estimating MVA in patients with MS [23]. The method is based on the time required for the initial TMG to decrease by 50% during diastole and is calculated from the deceleration slope of the early diastolic transmitral velocity profile obtained by continuous-wave Doppler [23]. According to the empirical formula, MVA is estimated as 220 divided by the PHT [23]. The main advantage of PHT is its simplicity and broad availability in routine clinical practice.

However, PHT has important limitations and should not be interpreted in isolation, particularly in complex hemodynamic settings. The method was originally validated in RMS, where it generally correlates reasonably well with invasive measurements in stable conditions [23]. Nevertheless, PHT is strongly influenced not only by the anatomical severity of stenosis but also by LA and LV compliance, transmitral flow, and associated valvular lesions. Conditions associated with altered LV relaxation or elevated LV diastolic pressure may shorten PHT and consequently lead to overestimation of MVA. Similarly, significant aortic regurgitation, tachycardia, AF may substantially affect measurement reliability [23].

A specific limitation of the PHT method should also be recognized in the early period following PMC. Immediately after the procedure, the abrupt increase in MVA is accompanied by acute changes in LA and LV compliance, as well as altered transmitral flow dynamics [23]. Consequently, PHT frequently overestimates the severity of residual MS and should not be considered a reliable surrogate for MVA during the early post-procedural period [23]. In this setting, direct planimetry, preferably by 2D or 3D TTE, represents the preferred method for assessing the immediate procedural result, while Doppler-derived measurements should be interpreted with caution until hemodynamic conditions have stabilized [23] (Table 3 and Table 4).

The limitations of PHT become even more pronounced in DMS. In elderly patients with DMS, reduced LV compliance and concomitant diastolic dysfunction are extremely common and frequently alter transmitral filling dynamics independently of valve anatomy. Moreover, the tunnel-like geometry of DMS differs substantially from the funnel-shaped RMS for which the method was originally developed. As a consequence, PHT often underestimates the true severity of MS in DMS and may produce discordant results compared with planimetry and mean TMG. Therefore, current evidence suggests that PHT should be used cautiously in DMS and always interpreted within an integrated multiparametric assessment [7,32].

### 4.5. Continuity Equation

The continuity equation represents an alternative Doppler-based method for estimating MVA in patients with MS, based on the principle of conservation of mass. According to this approach, the flow passing through the MV during diastole must equal the flow measured at another cardiac site, most commonly the left ventricular outflow tract (LVOT) [33]. MVA is therefore calculated by dividing stroke volume by the transmitral velocity-time integral obtained with continuous-wave Doppler [33].

Compared with PHT, the continuity equation is less influenced by LV and LA compliance and may provide a more reliable estimation of MVA in patients with altered diastolic properties or discordant TTE findings. This method may be particularly useful in DMS, where conventional Doppler-derived parameters often become unreliable [33].

However, the continuity equation also has important limitations. Accurate calculation requires precise measurement of LVOT diameter and Doppler signals, and small errors may significantly affect the final estimation of MVA [33]. In addition, the method assumes stable and equivalent forward stroke volume and is therefore unreliable in the presence of significant MR, aortic regurgitation, intracardiac shunts, or irregular rhythms. Reduced flow states may further complicate interpretation. Moreover, extensive annular calcification and acoustic shadowing may impair Doppler alignment and limit reproducibility in patients with severe MAC [33,34,35,36,37,38].

Overall, the assessment of DMS remains considerably more challenging than that of rheumatic MS because conventional TTE parameters were originally developed and validated in patients with rheumatic disease. Consequently, no single parameter should be considered definitive in DMS. A comprehensive multiparametric approach integrating valve morphology, Doppler-derived hemodynamics, three-dimensional TTE, and cCT is therefore recommended to avoid misclassification of disease severity and to optimize therapeutic decision-making.

### 4.6. Integrating Discordant Findings: A Practical Framework for Low-Gradient Severe MS and High-Gradient Non-Severe MS

Given that MVA and TMG reflect different aspects of MS (anatomical obstruction versus flow-dependent hemodynamics), their discordance is common in clinical practice, particularly in DMS, and should not be dismissed as measurement noise. Two discordant patterns are clinically relevant: low-gradient severe MS (MVA ≤ 1.5 cm^2^ with TMG < 5 mmHg) and high-gradient non-severe MS (MVA > 1.5 cm^2^ with TMG ≥ 5–10 mmHg). Critically, a low TMG must never be interpreted as reassurance of non-severe disease, since it may simply reflect a low-flow state superimposed on a genuinely small orifice.

When facing discordant findings, we propose a stepwise integration of the parameters discussed above rather than reliance on any single measurement. The suggestions are as follows:

- Re-verify MVA using at least two independent methods (2D/3D planimetry and continuity equation), prioritizing 3D-TOE and cCT in heavily calcified or tunnel-like DMS anatomy, where 2D planimetry and PHT are least reliable (Table 3 and Table 4).
- Correct for rhythm and heart rate, averaging TMG over ≥5 cycles in AF and accounting for the effect of tachy-/bradycardia on diastolic filling time.
- Characterize flow status: identify low-flow states (reduced stroke volume, LV systolic dysfunction, concomitant severe tricuspid or aortic disease) that may mask a low gradient despite severe anatomical stenosis, or high-flow/high-output states and significant concomitant MR that may inflate the gradient despite a non-severe orifice.
- Assess LV diastolic compliance, particularly in elderly DMS patients, since reduced compliance and diastolic dysfunction independently elevate filling pressures and can distort TMG-MVA correlation.
- Screen for concomitant valve disease (aortic stenosis/regurgitation, tricuspid regurgitation, mixed mitral disease), which alters transmitral flow dynamics and must be factored into interpretation.
- Consider stress TTE in equivocal or discordant cases to unmask flow-dependent gradient changes, although its diagnostic thresholds remain less validated in DMS than in RMS.

This integrated approach avoids both underestimation of severity in low-flow, low-gradient DMS and overestimation in high-flow or high-MR states, and should guide, together with symptoms and comorbidity burden, the decision to pursue further intervention.

### 4.7. From Anatomical Obstruction to Clinically Significant Disease: A Conceptual Framework

Grading MS severity has traditionally relied on anatomical thresholds of MVA, as reflected in the American Society of Echocardiography/European Association of Cardiovascular Imaging (ASE/EACVI) mild–moderate–severe classification. However, anatomical obstruction, hemodynamic burden, prognostic impact, and indication for intervention do not always align, and conflating these dimensions may lead to inappropriate clinical decisions, particularly in DMS and in patients with low-flow states. Anatomical obstruction refers strictly to the geometric reduction in the mitral orifice, best quantified by planimetry (2D/3D) or cCT, largely independent of loading conditions.

Hemodynamic burden reflects the functional consequence of this obstruction under a given flow state, captured by TMG and PHT, and is inherently flow-dependent. Prognostic implications depend on the interaction between anatomical severity, hemodynamic burden, and downstream consequences such as PH, atrial remodeling, and RV dysfunction, and are further modulated in DMS by the extensive comorbidity and frailty burden. Indication for intervention should not be based on anatomical MVA alone, but on the integration of symptoms, hemodynamic burden, and prognosis, together with procedural feasibility and risk—considerations that differ substantially between RMS, where PMC is often applicable, and DMS, where surgical or transcatheter options carry higher procedural risk. This conceptual distinction underlies the terminology adopted by the 2025 European Society of Cardiology (ESC) guidelines, which move away from a purely anatomical mild/moderate/severe classification toward the concept of clinically significant MS (MVA ≤ 1.5 cm^2^), a threshold intended to identify disease with therapeutic relevance rather than to grade anatomical severity per se (Table 2) [29].

### 4.8. Stress Echocardiography

Stress TTE plays an important role in the evaluation of patients with MS, particularly when symptoms appear disproportionate to resting TTE findings or when clinical assessment remains inconclusive [39]. Since TMGs are highly flow-dependent, patients with apparently moderate MS at rest may develop marked hemodynamic deterioration during exercise or stress conditions [39].

Exercise TTE is the preferred stress modality whenever feasible, as it provides a more physiological assessment of functional capacity and valvular hemodynamics [30,40,41]. During exercise, increases in heart rate and cardiac output may lead to a significant rise in TMG and sPAP, thereby unmasking latent hemodynamic severity not evident under resting conditions [42,43,44]. Exercise stress TTE is recommended in symptomatic patients with apparently moderate MS or when resting TTE findings are discordant with the clinical presentation, particularly when symptoms cannot be adequately explained by resting hemodynamic measurements [29]. Semi-supine bicycle exercise is generally the preferred modality, as it allows continuous acquisition of Doppler parameters throughout exercise, including TMG and sPAP, whereas treadmill exercise is less suitable because Doppler measurements are obtained only immediately after exercise [29]. In patients with AF, stress TTE remains feasible, although interpretation requires averaging multiple cardiac cycles and careful heart rate control because beat-to-beat variability may affect Doppler measurements [29]. Exercise-induced PH and marked increases in TMG identify patients with functionally significant MS despite apparently non-severe resting findings and may support consideration for intervention when integrated with symptoms and valve morphology. However, these thresholds have been primarily validated in RMS and should be interpreted with caution in DMS, where the hemodynamic response to exercise is less well established [29]. Contraindications to stress testing are consistent with standard exercise TTE recommendations and include unstable clinical conditions, uncontrolled arrhythmias, severe hypertension, or other situations in which exercise testing is considered unsafe [29].

Furthermore, current guidelines suggest that an exercise-induced mean TMG greater than 15 mmHg (18 mmHg with dobutamine infusion) or a sPAP exceeding 60 mmHg may identify patients with functionally severe MS and support consideration for intervention, particularly in symptomatic individuals [29,30]. It is important to emphasize that dobutamine stress TTE should not be regarded as interchangeable with exercise testing: unlike exercise, dobutamine increases heart rate and contractility without reproducing the physiological increase in preload and venous return that characterizes true exertion, a factor of particular relevance in a preload-sensitive lesion such as MS. This distinction is reflected in the differential gradient threshold adopted for dobutamine (18 mmHg) compared with exercise (15 mmHg), and dobutamine stress should therefore be reserved for patients unable to exercise, with results interpreted with additional caution. These thresholds are primarily derived from landmark observational studies evaluating the hemodynamic response to exercise or dobutamine stress rather than from randomized clinical trials. Accordingly, they should be regarded as supportive markers of hemodynamic significance rather than absolute intervention criteria. In routine clinical practice, stress TTE findings should always be interpreted within an integrated multiparametric assessment, taking into account symptoms, MVA, resting hemodynamics, valve morphology, pulmonary pressures, and overall procedural suitability [29,30].

Despite its clinical value, stress TTE has limitations, including variability related to exercise workload, AF, and operator dependency [45]. Moreover, standardized cutoff values remain less robust than resting TTE parameters [45]. Furthermore, resting and exercise-induced sPAP is age dependent and post-exercise sPAP has been shown to correlate inconsistently with symptom onset [46]. Nonetheless, an early increase in sPAP > 90% during exercise has been associated with adverse clinical implications [29].

Recent evidence further supports the prognostic role of exercise-induced PH in RMS. In a cohort of patients undergoing exercise stress TTE, sPAP increased markedly during exercise and emerged as an independent predictor of adverse clinical outcomes, including the need for percutaneous or surgical mitral valve intervention [47]. Furthermore, exercise-induced PH was shown to depend not only on TMG augmentation but also on LA volume, net atrioventricular compliance, and RV functional reserve, highlighting the complex hemodynamic interplay underlying symptom development in MS [47]. Moreover, exercise sPAP provided incremental prognostic value beyond resting measurements and MVA alone, supporting the use of stress TTE particularly in patients with discordant symptoms or apparently moderate MS at rest [47].

## 5. Prognostic Assessment in Mitral Valve Stenosis

Although TTE grading of MS severity relies primarily on anatomical and hemodynamic parameters, prognosis is determined by a broader set of markers reflecting the downstream consequences of chronic left atrial pressure overload, as well as the flow conditions under which severity is assessed. sPAP, estimated non-invasively from the peak tricuspid regurgitant velocity, remains one of the most established prognostic markers, reflecting the retrograde transmission of elevated LA pressure to the pulmonary circulation previously described and correlating with symptom burden and outcome independently of MVA [12,13,14]. Resting sPAP may nonetheless underestimate the true hemodynamic burden in patients with exercise-induced PH, supporting the role of stress echocardiography in selected symptomatic patients with non-severe resting findings. As pulmonary pressure overload persists, right ventricular function progressively declines; RV functional parameters, such as tricuspid annular plane systolic excursion or RV free wall strain, provide incremental prognostic information beyond sPAPalone, since RV impairment reflects the cumulative hemodynamic burden and predicts worse outcomes even when PASP is only moderately elevated.

Upstream of the pulmonary and right heart consequences of MS, left atrial function itself carries independent prognostic value. Reduced LA compliance amplifies pressure transmission for any given volume load (Section 3.2), and LA reservoir strain, a speckle-tracking-derived measure of atrial compliance, provides a quantitative and relatively load-independent assessment of this process, with emerging associations with symptom onset, development of atrial fibrillation, and post-intervention outcomes, potentially outperforming conventional LA diameter or volume. AF itself is both a consequence and a prognostic modifier of MS, reflecting chronic atrial pressure/volume overload and structural remodeling; beyond its recognized thromboembolic implications, AF independently complicates hemodynamic assessment and is associated with symptom progression and adverse outcomes, and should therefore be regarded as a prognostic marker in its own right rather than merely a measurement confounder.

Because gradient-based severity assessment is inherently flow-dependent, flow status itself carries prognostic weight and underlies much of the discordance discussed in Section 4.6. Net atrioventricular compliance, integrating both LA and LV compliance, captures this interaction more comprehensively than gradient or MVA alone, since reduced compliance amplifies the pressure response to a given transmitral flow and has shown prognostic value independent of anatomical MVA, particularly in borderline or discordant cases. Similarly, mitral valve resistance, calculated as the ratio between mean TMG and mitral flow rate, has been proposed as a flow-adjusted parameter potentially less influenced by transvalvular flow than TMG alone, and may help refine risk stratification in discordant MS, although its clinical validation remains more limited than that of MVA or TMG and it is not yet incorporated into routine practice or guideline-based thresholds. Taken together, these markers indicate that prognostic assessment in MS—particularly in DMS, where discordant and low-flow presentations are common—should extend beyond anatomical MVA and resting gradient to incorporate right heart hemodynamics, atrial function and rhythm, ventricular-atrial compliance, and flow-adjusted parameters into a comprehensive risk evaluation.

## 6. Mitral Valve Stenosis Assessment in Multivalvular Disease

Although the primary focus of this review is the multimodality assessment of MS, it is important to acknowledge that multivalvular heart disease is frequently encountered in both RMS and DMS (MAC-related) forms. A comprehensive discussion of concomitant valvular lesions is beyond the scope of this review; however, their recognition is clinically relevant because they may substantially influence the hemodynamic evaluation of MS, therapeutic decision-making, and overall prognosis. Therefore, a systematic assessment of associated valve disease should always be included in the comprehensive evaluation of these patients.

Specifically, in rheumatic disease, concomitant aortic valve involvement frequently reflects the diffuse nature of the rheumatic process, whereas degenerative aortic stenosis commonly coexists with MAC-related MS in elderly patients. In addition, significant functional tricuspid regurgitation is often present as a consequence of long-standing PH, right ventricular remodeling, and AF. The presence of associated valvular disease may influence hemodynamic assessment, timing of intervention, procedural planning, and long-term prognosis, highlighting the importance of Heart Team discussion [22,29].

## 7. Echocardiographic Scores for Therapeutic Decision-Making

### 7.1. Scores for Rheumatic Mitral Stenosis

Several TTE scoring systems have been developed over the past decades to improve patient selection and procedural planning for PMC in RMS, reflecting the strong influence of valve morphology on immediate and long-term procedural outcomes (Table 2) [48].

Among these, the Wilkins score remains the most widely used and historically validated model. Introduced in the 1980s, this semiquantitative score evaluates four anatomical components—leaflet mobility, leaflet thickening, valvular calcification, and subvalvular involvement—each graded from 1 to 4, generating a total score ranging from 4 to 16 [49]. The score was initially validated at the Massachusetts General Hospital in a relatively small cohort of 22 patients undergoing PMC. Traditionally, a score ≤ 8 has been associated with favorable anatomical suitability and higher likelihood of successful PMC, whereas scores ≥ 10 are generally linked to lower procedural success, increased risk of significant post-procedural MR, and worse long-term outcomes [49]. However, during the early procedural experience in the United States, the correlation between Wilkins score and post-procedural MVA gain was not consistently demonstrated [48]. Moreover, in a separate report published in the same year as the original description of the score, the authors specifically acknowledged that the Wilkins classification was not able to reliably predict the occurrence of severe post-procedural MR [48]. Despite its widespread adoption, the Wilkins score therefore has several important limitations. In particular, it provides only limited assessment of commissural morphology and does not adequately characterize commissural calcification, which is now recognized as one of the principal determinants of effective commissural splitting during balloon dilation [48]. Furthermore, the score does not fully account for asymmetric leaflet remodeling, heterogeneous calcium distribution, or the complexity of subvalvular fibrosis, all of which may significantly influence procedural feasibility and outcomes [48]. Nevertheless, subsequent evidence from the North American Multicenter Inoue Balloon Registry demonstrated that patients with Wilkins scores >10 had a significantly lower probability of achieving optimal procedural success, reinforcing the overall prognostic value of anatomical scoring despite its intrinsic limitations [50].

As understanding of MV anatomy and procedural mechanisms has evolved, several alternative and complementary scoring systems have been proposed. Particular attention has been directed toward commissural anatomy, since complete commissural opening represents the primary mechanism underlying successful PMC [48]. Commissural calcification, especially bilateral or extensive calcification, has consistently emerged as a strong predictor of suboptimal valve opening, residual stenosis, and increased risk of severe post-procedural MR [48]. Accordingly, dedicated commissural calcium scores have been developed to better stratify procedural suitability beyond the traditional Wilkins classification. Similarly, other models such as the revisited TTE score proposed by Nunes et al. integrated additional variables including severe subvalvular disease, reduced MVA, and asymmetrical commissural involvement, demonstrating superior predictive performance compared with the conventional Wilkins score, particularly in patients with intermediate anatomical complexity, who can be reclassified [51].

The Cormier score is an anatomical classification system used to assess the suitability of patients with RMS for PMC. The score evaluates key morphological features including AML mobility, leaflet calcification, and the extent of subvalvular involvement [48]. Based on these characteristics, patients are stratified into three groups: Group 1 identifies patients with favorable anatomy and high likelihood of procedural success; Group 2 includes patients with preserved leaflet mobility but significant subvalvular disease; and Group 3 characterizes patients with extensive calcification and unfavorable valve morphology, typically associated with lower procedural success and poorer outcomes. The Cormier classification therefore provides an additional tool for anatomical risk stratification and therapeutic decision-making in patients considered for PMC [48].

The increasing adoption of 3D-TTE has further refined morphological assessment in RMS. Indeed, in 2010, Anwar et al. generated a real time 3D TTE score in 17 patients and further validated in 74 young patients with a mean Wilkins score of 9 undergoing PMC at Al-Hussein University Hospital (Egypt) [52]. Higher weight was given to commissural/juxta commissural calcifications. Furthermore, an acquisition protocol was suggested to optimize the score’s yield, entailing multibeat acquisition, zoom 3D images and full 3D apical images to evaluate the chordae through their length [52].

Beyond guiding procedural feasibility, anatomical scoring also underpins an evolving therapeutic paradigm concerning the timing of intervention in RMS. In patients classified as having favorable anatomy—typically corresponding to a Wilkins score ≤ 8 [49] or Cormier Group 1 [48]—there is growing interest in considering early PMC in apparently asymptomatic individuals who present high-risk features, rather than adhering strictly to a watchful-waiting strategy reserved for symptomatic or hemodynamically severe disease. Evidence from the North American Multicenter Inoue Balloon Registry supports the association between favorable anatomical scoring and higher procedural success, reinforcing the rationale for considering intervention earlier in appropriately selected patients [50]. Proposed high-risk features supporting consideration of early PMC include exercise-induced PH or a marked exercise-induced rise in TMG, which has been shown to independently predict the need for percutaneous or surgical mitral valve intervention [47], as well as elevated resting or exercise pulmonary artery pressures identified through the guideline-based thresholds discussed above [29,30]. Because favorable anatomy is a prerequisite for a low-risk percutaneous approach, this early-intervention strategy is inherently dependent on accurate anatomical stratification by the scoring systems described above [48,49,51], and is not interchangeable with the accompanying stress TTE assessment, which instead identifies functional/hemodynamic—rather than anatomical—high-risk features. It should be emphasized, however, that this remains an evolving concept rather than an established standard of care: current recommendations for early PMC in truly asymptomatic patients are based largely on expert consensus and observational data rather than randomized controlled trials, and the long-term benefit of early intervention compared with careful surveillance has not been definitively established.

Overall, although several TTE scoring systems have improved risk stratification and procedural planning in RMS, no single model adequately captures the full anatomical and hemodynamic complexity of the disease (Table 3). Consequently, contemporary therapeutic decision-making increasingly relies on an integrated multimodality and multiparametric approach combining valve morphology, commissural anatomy, subvalvular remodeling, pulmonary hemodynamics, and clinical status to optimize patient selection for PMC (Table 5).

### 7.2. Scores for Mitral Annular Calcification

**Figure 4:**
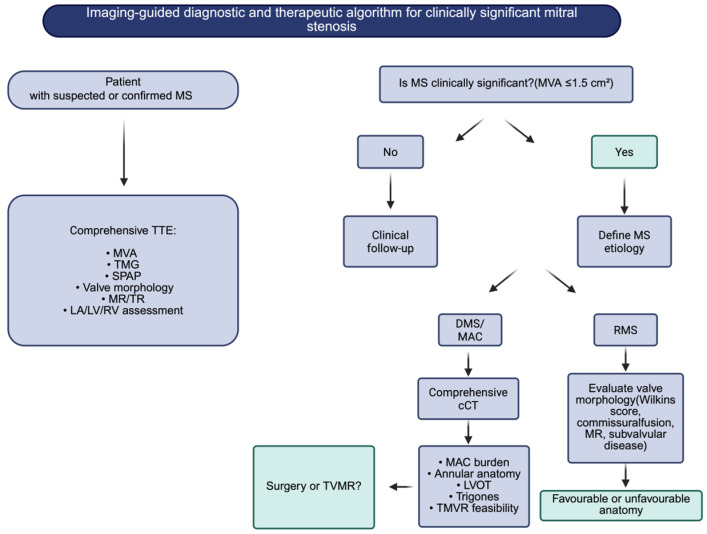
Imaging-guided diagnostic and therapeutic algorithm for clinically significant mitral stenosis. Abbreviations: cCT: cardiac computed tomography; DMS: degenerative mitral stenosis; LA: left atrium; LV: left ventricle; LVOT: left ventricular outflow tract; MAC: mitral annular calcification; MR: mitral regurgitation; MS: mitral stenosis; MVA: mitral valve area; RMS: rheumatic mitral stenosis; RV: right ventricle; SPAP: systolic pulmonary artery pressure; TMG: mean transmitral gradient; TTE: transthoracic echocardiography; TMVR: transcatheter mitral valve replacement.

**Figure 5:**
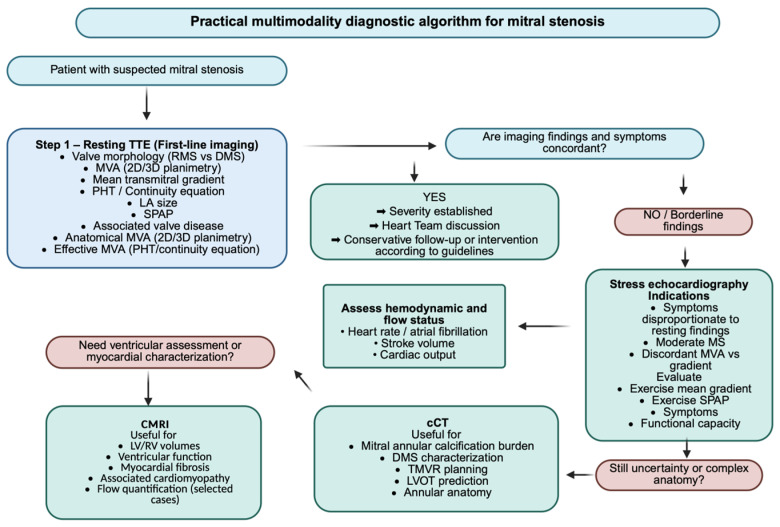
Practical multimodality diagnostic algorithm integrating echocardiography, stress echocardiography, cardiac CT, and cardiac magnetic resonance in the evaluation of mitral stenosis. Abbreviations: cCT: cardiac computed tomography; CMR: cardiac magnetic resonance; DMS: degenerative mitral stenosis; LA: left atrium; LV: left ventricle; LVOT: left ventricular outflow tract; MAC: mitral annular calcification; MR: mitral regurgitation; MS: mitral stenosis; MVA: mitral valve area; PH: pulmonary hypertension; PHT: pressure half-time; PMC: percutaneous mitral commissurotomy; RMS: rheumatic mitral stenosis; SPAP: systolic pulmonary artery pressure; TMVR: transcatheter mitral valve replacement; TTE: transthoracic echocardiography.

Several contemporary classifications have been proposed to improve the standardized assessment of MAC severity and its implications for mitral valve interventions. Among these, Xu et al. developed a novel multiparametric cCT-based MAC score designed to provide a comprehensive anatomical characterization of MAC beyond simple qualitative assessment [53]. The score integrates five major cCT-derived components, including Agatston calcium burden, circumferential extension of calcification, trigone involvement, myocardial infiltration, and extension toward the left ventricular outflow tract or aorto-mitral curtain, generating a cumulative score ranging from 2 to 12 [53]. In the original validation study, higher MAC scores were significantly associated with increased operative complexity, longer procedural and cross-clamp times, higher in-hospital complication rates, and increased mortality following mitral valve surgery, highlighting the value of systematic cCT-based anatomical assessment in predicting surgical risk [53]. Subsequently, Okushi et al. further validated the prognostic implications of this classification in patients undergoing MV surgery by stratifying MAC severity into severe (score 9–12) and non-severe (score 2–8) categories [54]. Although in-hospital mortality did not differ significantly between groups, severe MAC was associated with significantly higher long-term all-cause mortality, confirming the prognostic utility of structured MAC severity stratification [54]. More recently, Guerrero et al. proposed a broader grading and staging framework integrating both anatomical and clinical features to guide treatment selection in patients with MAC undergoing surgical or transcatheter mitral valve interventions. This classification introduced the concept of favorable (“green light”), challenging (“yellow light”), and prohibitive (“red light”) anatomy based on multimodality imaging findings, comorbidities, and procedural feasibility, reflecting the increasing transition from purely anatomical classification systems toward comprehensive multimodality and patient-centered therapeutic decision-making [28] (Figure 4 and Figure 5, Table 6).

**Table 6:** Scores for the assessment of MV stenosis.

Score	Imaging Modality	Main Variables	Main Strengths	Main Limitations	Clinical Impact
Wilkins score [49]	2D-TTE	Leaflet mobility, leaflet thickening, calcification, subvalvular involvement (4–16)	Historically validated; widely used	Poor commissural assessment; limited prediction of post-PMC MR	Standard anatomical selection for PMC
Nunes score [51]	2D-TTE	Wilkins variables + commissural asymmetry + severe subvalvular disease	Better prediction in intermediate anatomy	More complex; less routinely used	Improved procedural risk stratification
Cormier score [48]	TTE + fluoroscopy	AML mobility, calcification, subvalvular disease	Simple anatomical classification	Limited hemodynamic integration	Anatomical suitability for PMC
Anwar 3D score [52]	3D-TTE	Commissural morphology, leaflet disease, subvalvular involvement	Improved spatial and commissural assessment	Limited validation cohorts	Advanced pre-procedural evaluation
MAC score [53]	cCT	Calcium burden, circumferential MAC extension, trigones, myocardial infiltration, LVOT extension	Predicts surgical complexity and outcomes	cCT-dependent	Surgical/transcatheter risk stratification
MAC classification [54]	cCT	Severe vs. non-severe MAC based on Xu score	Prognostic validation of MAC severity	Derived from surgical population	Long-term prognostic assessment
MAC staging [28]	Multimodality imaging	Anatomical complexity + clinical factors	Integrates anatomy, feasibility, and comorbidities	Less quantitative	Guidance for surgical vs. transcatheter intervention

Abbreviations: AML: anterior mitral leaflet, cCT: cardiac computed tomography, LVOT: left ventricular outflow tract, MAC: mitral annular calcification, MR: mitral regurgitation, MS: mitral stenosis, PBMV: percutaneous balloon mitral valvuloplasty, PH: pulmonary hypertension, PMC: percutaneous mitral commissurotomy, RMS: rheumatic mitral stenosis, TOE: transesophageal echocardiography, TTE: transthoracic echocardiography.

## 8. The Role of Multimodality Imaging in the Evaluation of Mitral Valve Stenosis

Despite the central role of TTE in the diagnosis and grading of MS, no single imaging modality is sufficient to comprehensively evaluate all anatomical, functional, and procedural aspects of the disease. Contemporary management therefore relies on a multimodality imaging approach in which TTE, TOE, cCT, and cardiac magnetic resonance (CMR) provide complementary information according to the clinical scenario and therapeutic objective [22,29].

TTE remains the first-line imaging modality and represents the cornerstone of MS assessment. Nevertheless, it has intrinsic limitations. Heavy calcification, acoustic shadowing, poor acoustic windows, and the complex 3D anatomy of the MV may reduce diagnostic accuracy, particularly in DMS [22]. Under these circumstances, cCT has emerged as the most important complementary imaging modality [55].

In patients with MAC-related MS, cCT provides superior spatial resolution and allows accurate characterization of the extent, thickness, and circumferential distribution of MAC, features that cannot always be adequately appreciated by TTE [17,24]. In addition, cCT enables precise assessment of leaflet calcification, involvement of the fibrous trigones, and extension toward the LVOT, all of which are critical determinants of procedural feasibility and risk stratification [17,24,29]. The Heart Valve Collaboratory cCT scoring system, firstly created for the aortic valve, further enhances anatomical evaluation by integrating calcium burden, annular involvement, leaflet extension, and LVOT anatomy into a comprehensive assessment that supports therapeutic decision-making [56].

The contribution of cCT becomes even more relevant when transcatheter mitral interventions are considered. In patients undergoing transcatheter mitral valve replacement (TMVR), cCT represents the reference imaging modality for annular sizing, prosthesis selection, prediction of neo-LVOT obstruction, and procedural simulation [56]. Three-dimensional reconstruction allows detailed evaluation of the mitral annulus and its relationship with adjacent cardiac structures, facilitating device selection and reducing the risk of procedural complications [17,24,29]. Therefore, while TTE remains indispensable for functional assessment, cCT provides the anatomical information required for contemporary procedural planning.

Although its role has traditionally been emphasized in DMS, cCT is increasingly recognized as a valuable complementary tool in RMS as well, particularly for procedural planning before PMC. cCT allows detailed volumetric characterization of commissural calcification, a key determinant of successful commissural splitting and post-procedural outcome that is not adequately captured by conventional TTE-based scoring systems such as the Wilkins score [48,49]. Beyond the commissures, cCT can further characterize the extent and distribution of subvalvular calcification and fibrosis, providing incremental information on subvalvular disease severity beyond conventional bidimensional (2D) and 3D TTE. In addition, cCT enables precise assessment of mitral annular anatomy and dimensions, which may inform procedural planning and risk stratification in anatomically complex or borderline cases considered for PMC. While this application of cCT remains less standardized than its established role in MAC and TMVR planning, it represents a growing area of interest for refining patient selection in RMS.

Among emerging technologies, photon-counting CT (PCCT) may further refine the anatomical assessment of MS. Compared with conventional CT, PCCT offers improved spatial resolution and reduced blooming artifact from calcium, potentially allowing more accurate quantification and localization of both commissural/subvalvular calcification in RMS and annular/LVOT calcification in DMS. These technical advantages could translate into more precise preprocedural risk stratification for both PMC and transcatheter mitral interventions. However, evidence supporting the use of PCCT in MS remains limited to early observational experience, and larger validation studies are needed before its incorporation into standardized imaging protocols.

Compared with cCT, CMR has a more limited but complementary role in the routine evaluation of MS. Although it is not recommended as a first-line imaging modality, CMR provides highly reproducible quantification of biventricular volumes and systolic function and may be particularly useful when TTE findings are inconclusive or image quality is suboptimal [29]. Furthermore, CMR offers comprehensive assessment of atrial and ventricular remodelling and may identify concomitant myocardial disease that could contribute to symptoms independently of valve obstruction. Phase-contrast imaging also permits direct quantification of transmitral flow and can complement Doppler-derived hemodynamic assessment in selected patients, although its routine application for MVA estimation remains limited [29].

Beyond its role in valve assessment and procedural planning, multimodality imaging also contributes significantly to thromboembolic risk stratification and anticoagulation management in MS, complementing conventional rhythm-based assessment (i.e., presence and duration of AF). TOE remains the reference technique for the direct visualization of left atrial appendage (LAA) thrombus and spontaneous echo contrast (SEC), both of which are associated with increased thromboembolic risk and may influence anticoagulation decisions independently of rhythm status, including in patients with sinus rhythm. Beyond direct thrombus detection, indices of left atrial mechanical dysfunction—including reduced LAA emptying velocity and impaired left atrial reservoir strain on speckle-tracking echocardiography—have been proposed as complementary markers of atrial myopathy and thromboembolic risk, potentially identifying patients at increased risk even in the absence of overt AF or detectable thrombus. cCT, with its high spatial resolution and delayed-phase acquisition protocols, offers an alternative, less invasive method for the exclusion of LAA thrombus and may be particularly useful when TOE is contraindicated or poorly tolerated. Taken together, these imaging-derived parameters support a more individualized, mechanism-based approach to thromboembolic risk stratification in MS, complementing traditional rhythm-based criteria and potentially informing anticoagulation strategy in selected patients. However, their incorporation into standardized risk-stratification algorithms and anticoagulation decision-making remains an evolving area, and dedicated outcome data in MS populations are still limited.

Rather than competing with TTE, cCT and CMR should therefore be considered complementary imaging modalities that answer different clinical questions. TTE remains the reference technique for diagnosis, hemodynamic assessment, and follow-up, whereas cCT provides detailed anatomical characterization and has become indispensable for procedural planning in patients undergoing transcatheter mitral interventions, in both DMS and RMS. CMR complements this multimodality strategy through accurate assessment of cardiac chambers, ventricular function, and myocardial tissue characterization when additional information is required [57]. Accordingly, current recommendations advocate a patient-tailored multimodality imaging approach, in which each imaging technique contributes specific information to optimize diagnosis, risk stratification, procedural planning, and therapeutic decision-making in patients with MS [22,29].

## 9. Current Controversies and Knowledge Gaps

Although TTE remains the cornerstone of MS assessment, the preceding sections have highlighted that several aspects of its clinical application are supported by evidence of markedly different strength depending on aetiology and imaging modality. This section synthesizes the main areas of ongoing controversy and unresolved evidence gaps, with particular emphasis on the assessment of DMS, the role of stress TTE, and the incremental value of cCT and CMR in clinical decision-making.

### 9.1. DMS: An Extrapolated Rather than a Validated Diagnostic Pathway

The most consistent controversy emerging from this review concerns the diagnostic framework applied to DMS. Virtually every parameter discussed—planimetry, mean TMG, PHT, and the continuity equation—was developed and validated in RMS, and its application to DMS relies on pathophysiological analogy rather than dedicated outcome data [23,27]. This has three practical consequences that remain unresolved. First, no MVA threshold has been validated against hard clinical endpoints specifically in DMS; the 2025 ESC guidelines circumvent this gap by proposing a single “clinically significant” cut-off (MVA ≤ 1.5 cm^2^) rather than a graded severity classification, implicitly acknowledging that the mild/moderate/severe stratification used in RMS cannot be transposed with confidence [29]. Second, anatomical and hemodynamic parameters may diverge in either direction in DMS—TMG frequently underestimates severity because of the non-flow-accelerating, tunnel-like geometry of MAC, while PHT may simultaneously overestimate it because of concomitant diastolic dysfunction [23,27]—yet no validated algorithm exists to adjudicate between discordant parameters, unlike the relatively established primacy of planimetry in RMS. Third, the multimodality MAC classification proposed by the Heart Valve Collaboratory, while a valuable step toward standardizing terminology, remains a consensus-based framework that has not yet been prospectively validated against symptom onset, heart failure hospitalization, or mortality [28]. Taken together, the diagnostic assessment of DMS should currently be regarded as a rapidly evolving, expert-driven field rather than an evidence-validated pathway equivalent to that available for RMS, and this distinction warrants explicit acknowledgment when translating imaging findings into treatment decisions.

### 9.2. Stress Echocardiography: A Promising but Incompletely Validated Tool

Stress TTE is increasingly recommended to unmask latent hemodynamic severity in patients with symptoms discordant from resting findings [29,39]. However, the strength of this recommendation should be qualified. The exercise-induced thresholds most commonly cited (mean TMG > 15 mmHg, sPAP > 60 mmHg) derive from a limited number of observational cohorts rather than randomized outcome trials and should therefore be regarded as supportive hemodynamic markers rather than validated intervention criteria [29,30]. Their applicability is further constrained in clinical practice by AF, which is common in MS and complicates standardization of exercise protocols and averaging of Doppler measurements [29], and by an inconsistent relationship between post-exercise sPAP and symptom onset, partly attributable to the influence of age on pulmonary hemodynamics [46]. Most importantly, these thresholds were derived almost exclusively from rheumatic cohorts; their extrapolation to DMS is currently unsupported by dedicated data, since the already attenuated flow-gradient relationship described may further blunt or distort the hemodynamic response to exercise in this population [29]. While recent evidence has strengthened the prognostic role of exercise-induced PH in RMS, showing incremental value over resting measurements and MVA [47], no equivalent outcome data exist for DMS. Stress echocardiography should therefore be considered a valuable but still maturing tool in RMS, and an area of unmet research need in DMS.

### 9.3. Incremental Value of Cardiac cCT and CMR: Complementary Rather than Hierarchical Roles

A further source of ambiguity concerns how cCT and CMR should be positioned relative to TTE in clinical decision-making. The evidence reviewed indicates that their incremental value differs substantially by modality and aetiology, and should not be interpreted as a simple hierarchy. cCT provides genuine, non-redundant diagnostic information in DMS—quantifying MAC burden, characterizing trigone and LVOT involvement, and enabling neo-LVOT prediction—information that directly determines candidacy for TMVR and cannot be reliably obtained by TTE or TOE alone [17,24,53,56]. Its role in RMS, by contrast, remains largely complementary, mainly reserved for cases with inadequate acoustic windows or before high-complexity PMC [22,29]. CMR occupies a narrower niche: it offers reproducible quantification of biventricular volumes, atrial remodelling, and myocardial tissue characterization, but no CMR-derived parameter has been validated as a primary criterion for grading MS severity in either aetiology, and direct comparative studies benchmarking CMR against planimetry or invasive hemodynamics in MS are lacking [29,57]. Consequently, current evidence does not support routine CMR use for severity grading; its incremental value is best realized in selected patients in whom TTE findings are inconclusive or additional myocardial characterization is clinically warranted. Importantly, no study to date has directly compared stress echocardiography, cCT, and CMR within the same decision-making pathway (e.g., timing of intervention in DMS), and the three modalities should therefore be regarded as complementary—addressing hemodynamic reserve, anatomical/procedural planning, and myocardial tissue characterization, respectively—rather than interchangeable or ordered by a fixed diagnostic hierarchy.

### 9.4. Future Research Priorities

Addressing these gaps will require: prospective, outcome-driven studies establishing MVA and hemodynamic thresholds specific to DMS rather than extrapolated from RMS; dedicated validation of exercise and dobutamine stress TTE protocols in DMS populations, accounting for the confounding effects of age, comorbidity, and AF; independent, multicentre validation of the Heart Valve Collaboratory MAC classification against hard clinical endpoints; and head-to-head comparative studies of cCT and CMR against TTE and invasive reference standards to better define their incremental diagnostic and prognostic value across both aetiologies of MS.

## 10. Mitral Valve Disease in Special Population

**Figure 6:**
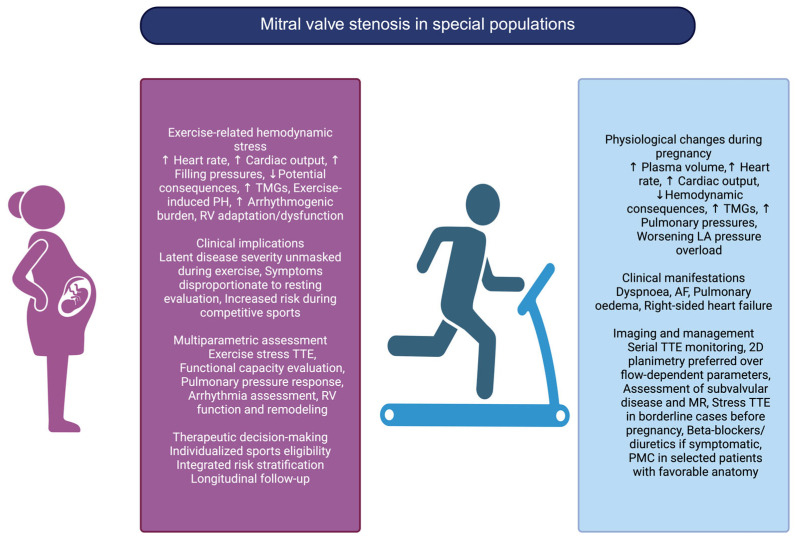
Schematic overview of the hemodynamic and clinical implications of MS in special populations, including pregnancy and competitive athletes. Abbreviations: AF: atrial fibrillation, LA: left atrial, MR: mitral regurgitation, MS: mitral stenosis, PAP: pulmonary artery pressure, PH: pulmonary hypertension, PMC: percutaneous mitral commissurotomy, RMS: rheumatic mitral stenosis, RV: right ventricular, TMG: transmitral mean gradient, TTE: transthoracic echocardiography.

Particular considerations should also be given to special patient populations, including pregnant women and competitive athletes, in whom MV disease may have unique hemodynamic and clinical implications (Figure 6) [58]. Pregnancy is associated with substantial physiological increases in plasma volume, heart rate, and cardiac output, which may significantly exacerbate TMGs and PH in women with MS, even when resting disease severity appears moderate. As a result, previously asymptomatic patients may develop worsening dyspnoea, AF, pulmonary oedema, or right-sided heart failure during pregnancy [59]. In this setting, TTE plays a crucial role not only in preconception counselling and risk stratification but also in serial monitoring throughout gestation. Stress TTE may occasionally provide additional information in borderline cases before pregnancy, while PMC remains the preferred intervention in symptomatic patients with favourable anatomy when medical therapy fails [60].

Similarly, athletes with MV disease represent a unique subgroup in whom exercise-induced hemodynamic changes may unmask latent functional severity not evident at rest [61]. Intensive physical activity may lead to marked increases in TMGs, PAP, and atrial arrhythmogenic burden, particularly in patients with RMS or significant MR. Therefore, comprehensive multiparametric evaluation, including exercise stress TTE, is often required to assess functional capacity, exercise-induced PH, arrhythmias, and ventricular adaptation before clearance for competitive sports participation [61].

In both pregnancy and athletic populations, individualized risk stratification integrating symptoms, valve anatomy, exercise hemodynamics, pulmonary circulation, and rhythm status is essential to optimize clinical management and reduce adverse cardiovascular events.

## 11. Future Directions

**Figure 7:**
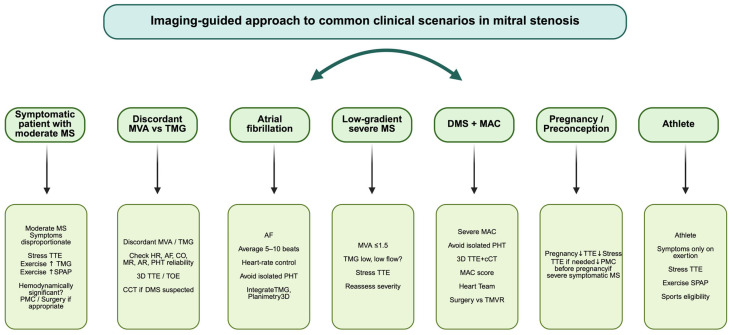
Imaging-guided approach to common clinical scenarios in mitral stenosis. Abbreviations: AF, atrial fibrillation; CO, cardiac output; cCT, cardiac computed tomography; DMS, degenerative mitral stenosis; HR, heart rate; LA, left atrium; LVOT, left ventricular outflow tract; MAC, mitral annular calcification; MR, mitral regurgitation; MS, mitral stenosis; MVA, mitral valve area; PMC, percutaneous mitral commissurotomy; PHT, pressure half-time; RMS, rheumatic mitral stenosis; SPAP, systolic pulmonary artery pressure; TMG, mean transmitral gradient; TMVR, transcatheter mitral valve replacement; TOE, transoesophageal echocardiography; TTE, transthoracic echocardiography.

Future research should focus on validating imaging parameters specifically for DMS and on developing standardized multimodality algorithms for patients with discordant anatomical and hemodynamic findings. Emerging imaging techniques, including advanced three-dimensional TTE, cCT for procedural planning, and quantitative CMR, may further improve diagnostic accuracy and patient selection for intervention. In addition, artificial intelligence has the potential to enhance automated image analysis, valve quantification, and risk stratification by integrating multimodality imaging with clinical data. However, these technologies require further prospective validation before being incorporated into routine clinical practice (Figure 7).

## 12. Conclusions

MV disease represents a highly heterogeneous pathological entity requiring comprehensive evaluation to optimize therapeutic decision-making. In RMS, TTE remains the cornerstone imaging modality for diagnosis, severity grading, and procedural planning, while stress TTE provides important incremental information by unmasking latent hemodynamic deterioration and exercise-induced PH not evident at rest [29]. Traditional anatomical scoring systems, including the Wilkins, Cormier, Nunes, and Anwar classifications, have significantly improved patient selection for PMC; however, their limitations have highlighted the need for a more integrated approach incorporating commissural anatomy, subvalvular remodelling, ventricular adaptation, and multimodality imaging findings [29].

Similarly, in degenerative MAC, contemporary cCT-based classifications and multiparametric MAC scores have demonstrated the importance of systematic anatomical characterization for predicting procedural complexity, surgical risk, and long-term prognosis. The increasing adoption of advanced imaging modalities, including 3D TTE, TOE, cCT, has progressively transformed MV assessment from a simplified morphology-based evaluation toward a comprehensive multimodality and patient-centred strategy [30].

Importantly, contemporary management of MV disease should no longer rely on isolated anatomical or hemodynamic parameters alone, but rather on an integrated multiparametric assessment combining valve morphology, commissural involvement, calcific burden, pulmonary circulation, LA and LV remodelling, procedural feasibility, and patient-specific clinical factors. This evolution reflects the broader transition toward precision cardiovascular medicine, where imaging is increasingly used not only for diagnosis but also for individualized risk stratification, procedural planning, and therapeutic guidance. Future advances in multimodality imaging, computational modelling, and imaging-guided intervention planning will likely further refine patient selection and improve outcomes in both rheumatic and degenerative MV disease. Furthermore, the value of multimodality imaging lies not in replacing TTE, but in integrating complementary information from different imaging techniques whenever conventional parameters are inconclusive or potentially misleading. The future of mitral stenosis assessment will therefore depend less on identifying a single “best” imaging modality and more on understanding how and when to combine TTE, TOE, stress TTE, cCT, and CMR to achieve accurate diagnosis, optimize patient selection, and guide individualized therapeutic decision-making.

## Figures and Tables

**Table 1:** Comparison between rheumatic and degenerative MS.

Feature	RMS	DMS
Typical population	Younger patients; endemic areas.	Elderly patients with multiple comorbidities.
Main mechanism	Commissural fusion.	MAC.
Valve morphology	Leaflet thickening and subvalvular fibrosis.	Annular and basal leaflet calcification.
Commissural involvement	Typical.	Usually absent.
Valve geometry	Funnel-shaped.	Tunnel-like.
Calcification pattern	Commissural/leaflet calcification in advanced stages.	Extensive annular calcification.
TMG	Usually reflects stenosis severity.	Often influenced by LV diastolic dysfunction.
Planimetry	Usually feasible.	Frequently challenging.
PHT	Relatively reliable.	Often unreliable.
Role of stress TTE	Identifies exercise-induced PH.	Useful in discordant low-gradient cases.
Role of cCT	Adjunctive role.	Essential for calcium burden and procedural planning.
Preferred intervention	PMC.	Surgery or transcatheter intervention.
Main scoring systems	Wilkins, Cormier, Nunes, Anwar.	cCT-based MAC scores.
Main prognostic factors	PH, RV dysfunction, commissural calcification.	Calcium burden, LVOT extension, frailty.

Abbreviations: cCT: cardiac computed tomography, DMS: degenerative mitral stenosis, LV: left ventricle, MAC: mitral annular calcification, PH: pulmonary hypertension, PMC: percutaneous mitral commissurotomy, RMS: rheumatic mitral stenosis, RV: right ventricle, TTE: trans-thoracic echocardiography.

**Table 2:** Comparison between the ASE/EACVI and 2025 ESC classifications of MS severity.

ASE/EACVI Recommendations [22]	2025 ESC Guidelines [29]
Mild (MVA > 2.5 cm^2^)	No equivalent category
Moderate (MVA 1.6–2.5 cm^2^)	No equivalent category
Severe (MVA ≤ 1.5 cm^2^)	Clinically significant MS: MVA ≤ 1.5 cm^2^. This threshold reflects the level of stenosis at which the disease is generally considered to have clinical and therapeutic relevance, warranting further evaluation based on symptoms, hemodynamic consequences, and suitability for intervention.

Abbreviations: ASE/EACVI: American Society of Echocardiography/European Association of Cardiovascular Imaging, MS: mitral stenosis, MVA: mitral valve area.

**Table 3:** Comparison of the main TTE methods for MVA assessment.

Method	Principle	Main Advantages	Main Limitations/Pitfalls	Best Clinical Setting
2D Planimetry	Direct anatomical tracing of the mitral valve orifice	Reference standard for anatomical MVA assessment; relatively independent of flow and loading conditions; particularly reliable in RMS	Operator dependent; requires correct imaging plane; limited by poor acoustic window, heavy calcification, acoustic shadowing, AF, and irregular valve geometry; may overestimate MVA if the imaging plane is oblique	First-line method in rheumatic MS; should always be integrated with Doppler findings
3D Planimetry (TTE/TOE)	Direct en-face visualization with multiplanar reconstruction	More accurate identification of the smallest orifice; reduces geometric assumptions and plane misalignment; particularly useful in DMS	Requires dedicated equipment and expertise; image quality dependent	Complex anatomy, heavily calcified valves, DMS, discordant findings
PHT	Estimation of MVA from transmitral deceleration slope (220/PHT)	Simple, rapid, widely available, extensively validated in rheumatic MS	Strongly influenced by LA/LV compliance, heart rate, AF, significant AR, MR, altered loading conditions, and early post-PMC hemodynamic changes; unreliable in DMS	Stable RMS when hemodynamic conditions are preserved; should not be used in isolation
Continuity Equation	Conservation of flow using LVOT stroke volume and transmitral VTI	Less dependent on LA/LV compliance than PHT; useful when Doppler parameters are discordant	Requires accurate LVOT measurements; unreliable in significant MR, AR, intracardiac shunts, irregular rhythm, or low-flow states; technically demanding	Selected patients with discordant findings or altered diastolic properties
Mean TMG	Doppler-derived hemodynamic assessment of transmitral pressure gradient	Easy to obtain; reflects functional hemodynamic severity; useful for follow-up and stress echocardiography	Highly flow dependent; influenced by heart rate, cardiac output, AF, MR, LV compliance, and loading conditions; should not be considered a direct measure of anatomical MVA	Integrated assessment together with planimetry and clinical findings

Abbreviations: AF: atrial fibrillation; AR: aortic regurgitation; DMS: degenerative mitral stenosis; LA: left atrial; LV: left ventricle; LVOT: left ventricular outflow tract; MR: mitral regurgitation; MS: mitral stenosis; MVA: mitral valve area; PMC: percutaneous mitral commissurotomy; PHT: pressure half-time; RMS: rheumatic mitral stenosis; TMG: mean transmitral gradient; TOE: transoesophageal echocardiography; TTE: transthoracic echocardiography; VTI: velocity–time integral.

**Table 4:** Diagnostic pitfalls in rheumatic versus degenerative mitral stenosis: a practical comparison.

Diagnostic Aspect	Rheumatic Mitral Stenosis (RMS)	Degenerative Mitral Stenosis (DMS)	Clinical Implication
Valve morphology	Commissural fusion, leaflet thickening, subvalvular involvement	Mitral annular calcification (MAC), basal leaflet calcification, usually no commissural fusion	Etiology determines suitability for PMC
MVA by 2D planimetry	Reference method when imaging plane is correct	May be inaccurate due to heavy calcification and acoustic shadowing	Consider 3D imaging and integrate with CCT
3D planimetry	Improves anatomical assessment	Particularly useful when 2D planimetry is limited by MAC	Preferred in complex anatomy
PHT	Valid in stable hemodynamic conditions	Frequently unreliable because of impaired LV compliance and MAC	Do not use as the sole parameter
TMG	Reflects hemodynamic severity but remains flow-dependent	Often overestimates severity because of reduced LV compliance and diastolic dysfunction	Interpret together with MVA and flow conditions
Stress TTE	Well validated for symptom evaluation and intervention selection	Limited evidence; thresholds less well established	Interpret cautiously in DMS
Role of cCT	Mainly complementary	Essential for MAC burden, annular anatomy, LVOT assessment, and procedural planning	Key modality before TMVR
Role of CMR	Selected cases	Selected cases	Assessment of ventricular remodeling and concomitant myocardial disease
PMC	Appropriate in favorable anatomy	Generally contraindicated because commissural fusion is absent	Etiology-driven treatment selection

**Table 5:** MS in multivalvular diseases.

Concomitant Lesion	Impact on MS Assessment	Practical Recommendation
MR	Invalidates the continuity equation (assumes no significant valvular regurgitation); may overestimate transmitral flow and gradient	Rely on planimetry (2D/3D) or pressure half-time; avoid continuity equation-derived MVA
AR	Invalidates the continuity equation (violates the assumption of equal LVOT and mitral stroke volume); may falsely elevate Doppler-derived gradients due to increased transmitral flow	Prefer planimetry; interpret pressure half-time with caution, as AR can shorten PHT independent of true MVA
AS	Reduced cardiac output/stroke volume may lower transmitral flow and underestimate TMG and PHT-derived severity (“low-flow” state)	Integrate MVA by planimetry; consider stress echocardiography if flow-dependence is suspected
TR/right heart dysfunction	May reduce LV preload and transmitral flow, potentially underestimating gradients; elevated right-sided pressures may confound sPAP interpretation	Interpret sPAP cautiously in the context of RV function; corroborate planimetry
Atrial fibrillation (associated valvular disease)	Beat-to-beat variability affects continuity equation, PHT, and TMG regardless of valve combination	Average measurements over multiple cardiac cycles (5–10) with similar preceding RR intervals

Abbreviations: AR, aortic regurgitation; AS, aortic stenosis; LV, left ventricle; LVOT, left ventricular outflow tract; MR, mitral regurgitation; MS, mitral stenosis; MVA, mitral valve area; PHT, pressure half-time; RV, right ventricle; sPAP, systolic pulmonary artery pressure; TMG, transmitral mean gradient.

## Data Availability

No new data were created or analyzed in this study.

## Abbreviations

The following abbreviations are used in this manuscript:

2D	two-dimensional
3D TOE	three-dimensional transoesophageal echocardiography
3D TTE	three-dimensional TTE
AF	atrial fibrillation
AML	anterior mitral leaflet
AR	aortic regurgitation
cCT	cardiac computed tomography
CMR	cardiac magnetic resonance
DMS	degenerative mitral stenosis
ESC	European Society of Cardiology
EACVI	European Association of Cardiovascular Imaging
LA	left atrial
LAA	left atrial appendage
LV	left ventricle
LVOT	left ventricular outflow tract
MAC	mitral annular calcification
MPR	multiplanar reconstruction
MR	mitral regurgitation
MS	mitral stenosis
MV	mitral valve
MVA	mitral valve area
PCCT	pthoton counting CT
PH	pulmonary hypertension
PHT	pressure half time
PMC	percutaneous mitral commissurotomy
PML	posterior mitral leaflet
PSAX	parasternal short-axis
RMS	rheumatic mitral stenosis
RV	right ventricular
SEC	spontaneous echo contrast
sPAP	systolic pulmonary artery pressure
TMG	trans-mitral gradient
TTE	echocardiography
TOE	trans-oesophageal echocardiography
TVMR	transcatheter mitral valve replacement
